# HIV case reporting in the countries of North Africa and the Middle East

**DOI:** 10.7448/IAS.17.1.18962

**Published:** 2014-05-19

**Authors:** Ivana Bozicevic, Gabriele Riedner, AliAkbar Haghdoost

**Affiliations:** 1 World Health Organization Collaborating Centre for HIV Surveillance, University of Zagreb School of Medicine, Croatia; 2 World Health Organization Regional Office for Eastern Mediterranean, Cairo, Egypt; 3 Regional Knowledge Hub, and WHO Collaborating Centre for HIV Surveillance, Institute for Futures Studies in Health, Kerman University of Medical Sciences, Kerman, Iran

**Keywords:** HIV, case reporting, HIV-related deaths, CD4 counts, surveillance, modes of transmission

## Abstract

The aim of the paper is to provide an overview of HIV case reporting data for the year 2011 from the countries of the World Health Organization Eastern Mediterranean Region (WHO EMR).

Fourteen countries provided data for the year 2011 and reported a total of 4263 HIV cases of which 66.8% were men. The highest number of reported HIV cases in men per 100,000 population was in Oman (5.8), Somalia (5.5) and Iran (3.3), while in women in Somalia (7.6), Oman (3.9) and Morocco (2.4).

In the majority of the countries, the most common reported mode of transmission was heterosexual. This could be due to under-reporting of male-to-male transmission and more frequent testing of men than women.

## Introduction

In 2011, the World Health Organization (WHO) estimated that 561,261 (414,711–802,311) people were living with HIV in the World Health Organization Eastern Mediterranean Region (WHO EMR) [1]. There was an estimated number of 82,994 (53,959–144,066) new HIV infections, while the number of AIDS deaths in 2011 was estimated at 38,418 (27,980–49,711).

A review of development and results of the national HIV surveillance systems of the countries of the WHO EMR found that the performance of HIV surveillance systems in several of the countries has improved in recent years but that the extent of HIV epidemics in the populations most at risk of HIV is still largely unknown in 10 countries [2].

HIV case reporting is one of the key components of HIV surveillance. WHO recommends that all countries strengthen their HIV case reporting systems in order to better estimate treatment and care needs and support public health policies for prevention of HIV epidemic [3]. In addition, the scale-up of HIV testing services has to be accompanied by an effective system of recording and reporting of those newly diagnosed with HIV. The WHO Office for the Eastern Mediterranean (EMRO) recommends that the countries collect the following variables on the form for reporting a case of HIV infection: sex, age, mode of HIV transmission and stage of HIV infection based on clinical, and wherever possible, immunological criteria.

The aim of this paper is to provide an overview of the most recent results of the HIV case reporting systems in the WHO EMR countries for the year 2011.

## Methods

To collect data on the reported cases of HIV infection and cases of deaths due to HIV in the year 2011, we used an annual HIV case reporting form that was sent in January 2013 from EMRO to the National AIDS Programmes (NAPs) in all 23 countries of the WHO EMR, namely Afghanistan, Bahrain, Djibouti, Egypt, Iran, Iraq, Jordan, Kuwait, Lebanon, Libya, Morocco, Oman, Pakistan, Qatar, Saudi Arabia, Somalia, Sudan, South Sudan, Syria, Tunisia, United Arab Emirates (UAE), Palestine and Yemen. The form sought the following data: cumulative number of HIV cases reported since beginning of reporting; number of HIV cases reported in the year 2011 by sex and mode of HIV transmission; proportion of reported HIV cases for whom data on CD4 counts at the time of HIV diagnosis were available and a proportion of HIV cases that had CD4 counts less than 350 cells/mm^3^ at the time of HIV diagnosis. To calculate the number of reported cases in 2011 in men and women per 100,000 population we used the UN population statistics [4]. Data provided in the HIV case reporting form were entered in Microsoft Excel and analyzed. We also present the data on the estimated number of people living with HIV, which are provided by UNAIDS and obtained using Estimation and Projection Package and Spectrum [5].

## Results

For the year 2011, 14 countries (Afghanistan, Iran, Jordan, Kuwait, Lebanon, Morocco, Oman, Palestine, Saudi Arabia, Sudan, South Sudan, Syria, Tunisia and UAE) provided data on reported HIV cases; 12 of these countries provided data on the cumulative number of HIV cases reported since the beginning of reporting while 10 countries provided data on reported cases of HIV-related deaths in 2011.

In total, 14 countries reported 4263 HIV cases in 2011, and of these 66.8% were men. Table 1 shows the number of reported HIV cases in men and women by the probable mode of HIV transmission, and only in Sudan and Somalia the information on the mode of transmission is not available. The highest absolute number of reported male cases in 2011 was in Iran (*n*=1247) while the number of cases per 100,000 male population was the highest in Oman (5.8) followed by Somalia (5.5) and Iran (3.3). Heterosexual sex was the most common reported mode of HIV transmission in men in the following countries: Tunisia (44.4%), UAE (50.0%), Syria (54.5%), Jordan (66.7%), Morocco (81.9%), Kuwait (100%) and Palestine (100%). Injecting drug use (IDU) was the most common mode of transmission in men in Iran (73.5%) and Afghanistan (60.2%). Unknown mode of transmission was the most prevalent in men in Oman (35.8%) and Saudi Arabia (60.9%). Male-to-male sex was reported as the most frequent mode of HIV transmission only in Lebanon (50.0%).

**Table 1:** Number of HIV cases reported by the NAPs in men and women in 14 countries of the World Health Organization Eastern Mediterranean Region and the estimated number of people living with HIV, 2011 data

	Number of HIV cases reported as IDU	Number of HIV cases reported as MSM	Number of HIV cases reported as heterosexual	Number of HIV cases reported as being due to MTC	Number of HIV cases reported as being due to receipt of blood and blood products	Number of HIV cases whose mode of HIV transmission is unknown	Total number of HIV cases reported	Rate per 100,000 population	Cumulative number of HIV cases reported since the beginning of HIV case reporting	Estimated number of people living with HIV^a^
	
	Male/female		Male/female	Male/female	Male/female	Male/female	Male/female	Male/female	Total	Total
Afghanistan^b^	50/2	1	13/7	0/0	0/0	19/15	83/24	0.6/0.2	1367	4000 (1500–12,000)
Iran	916/32	NA	99/241	38/24	0/0	194/44	1247/341	3.3/0.9	24,651	65,000 (48,000–89,000)
Jordan	0/0	3	8/5	0/0	1/0	0/0	12/5	0.4/0.2	248	NA
Kuwait	0/0	0	21/4	0/0	0/0	0/0	21/4	1.2/0.3	NA	NA
Lebanon^c^	1/0	24	23/2	0/0	0/1	0/0	48/3	2.2/0.1	NA	NA
Morocco	8/1	35	303/339	10/15	2/0	12/25	370/380	2.4/2.4	9352	28,000 (21,000–37,000)
Oman	5/0	22	30/28	4/6	0/0	34/11	95/45	5.8/3.9	1353	NA (2500–5100)^d^
Palestine	0/0	0	2/3	0/1	0/0	0/0	2/4	0.1/0.2	72	NA
Saudi Arabia	46/0	5	73/29	1/3	0/0	195/42	320/74	2.1/0.6	3429	NA
Somalia	NA	NA	NA	NA	NA	NA	264/369	5.5/7.9	3641	31,000 (21,000–47,000)
Sudan	NA	NA	NA	NA	NA	NA	239/114	NA	1450	NA (54,000–84,000)g
Syria	0/0	6	30/14	0/0	5/0	14/0	55/14	0.5/0.1	762	NA
Tunisia	3/0	3	20/27	0/1	0/0	19/0	45/28	0.9/0.5	1706	2000 (1200–3300)
United Arab Emirates	1/6	3	23/6	1/1	0/1	18/3	46/11	0.8/0.4	935	NA

a Estimates of the number of people living with HIV are available from UNAIDS (http://www.unaids.org/en/dataanalysis/knowyourepidemic/epidemiologypublications/).

b 117 male and female HIV cases were reported in Afghanistan, but for 10 cases the information on sex was missing.

c 109 male and female HIV cases were reported in Lebanon but only for 51 cases the information on mode of transmission and sex was available.

d For Oman and Sudan, only lower and higher confidence bounds of the estimates are available.

NA=not available; IDU=injecting drug use; MSM=men who have sex with men; MTC=mother-to-child transmission.

The highest absolute number of HIV cases in women in 2011 was reported in Morocco (*n*=380), while the rate of reported cases (per 100,000) was the greatest in Somalia (7.6), Oman (3.9) and Morocco (2.4) (Table 1). The most common transmission category in women in all the countries was heterosexual with the exception of Afghanistan and Saudi Arabia where the unknown mode of transmission predominated (62.5 and 56.8%, respectively). IDU cases in women were reported only in Iran (*n*=32), Afghanistan (*n*=2) and Morocco (*n*=1).

Only in Morocco, Somalia and Palestine the male-to-female ratio in the reported HIV cases in 2011 was lower than one (0.97:1; 0.7:1 and 0.5:1, respectively) while in all the other countries, the number of reported male cases substantially outnumbers those in females, ranging from 1.6:1 in Tunisia to 5.3:1 in Kuwait. The male-to-female ratio is particularly striking in Lebanon (16:1). However, in Lebanon the information on gender was missing for 53% of reported HIV cases.

Since the beginning of reporting until the end of 2011, 48,966 HIV cases were reported from 12 countries listed in Table 1 and of these 79.4% were men (data not shown in the table). In men, the highest cumulative number of reported cases was in Iran (*n*=22,358) while in women this was in Morocco (*n*=2931).

The estimated total number of people living with HIV in 2011 ranged from 2000 (1200–3300) in Tunisia to 65,000 (48,000–89,000) in Iran [6]. Although not directly comparable, of note is a considerable difference between the cumulative number of HIV cases reported and the estimated number of people living with HIV, particularly in Sudan and Somalia.

Information on CD4 counts at the time of HIV diagnosis in cases reported were available from NAPs in six countries (Jordan, Morocco, Oman, Palestine, Syria and Tunisia). The completeness, defined as a proportion of reported HIV cases who had CD4 count data reported within three months of HIV diagnosis out of all newly reported HIV cases, was reported as 100% in Morocco, while it was low in all other countries (6%, Syria; Palestine, 17%; Jordan 18%, Tunisia, 48%; Oman 67%). The proportion of cases that had CD4 counts of less than 350 cells/mm^3^ (the absolute number of these cases is shown after a proportion) was the highest in Palestine (100%, *n*=1), followed by Oman (64%, *n*=60), Tunisia (59%, *n*=21), Jordan (58%, *n*=2), Morocco (55%, *n*=412) and Syria (25%, *n*=1). As can be seen, in Syria and Palestine data on the proportion of cases that had CD4 counts less than 350 cells/mm^3^ are based on a very small number of cases.

Iran, Jordan, Kuwait, Palestine, Oman, Saudi Arabia, Somalia, Syria, Tunisia and UAE were able to report cases of HIV-related deaths in 2011. These 10 countries reported a total of 568 HIV-related deaths. However, 73.6% of all these cases were reported from Iran. Of the total 568 reported deaths, 82.7% were men.

Since the beginning of HIV-related deaths reporting, the highest number of deaths was reported in Iran (*n*=4777) followed by Oman (*n*=811). Iran contributed with 65.7% in all cases of HIV-related deaths reported in these 10 countries since the beginning of reporting, which shows the scale of under-reporting of HIV-related deaths across the region.

## Discussion

The rate of reported HIV cases varies widely among the observed countries.

The number of HIV cases reported per 100,000 male population in 2011 was the highest in Oman, followed by Somalia and Iran, while in women the rates were the highest in Somalia, Oman and Morocco. While the HIV epidemic in Iran, Somalia and Morocco has been relatively well characterized, very little is known about the HIV epidemic in Oman [7–10]. The case reporting data show an urgent need to establish better functioning HIV surveillance systems in particular in all Gulf Cooperation Council countries.

A high male-to-female ratio in reported HIV cases in majority of the countries described in this review is an important finding and could be due to male-to-male sexual transmission or HIV transmission that occurs among IDUs of whom most are male. However, due to presence of high-risk sexual behaviours in IDUs it would be expected that transmission of HIV is seen in their female partners [11]. Though the recent integrated bio-behavioural surveys show a concentrated HIV epidemic in MSM in a number of countries in the region (Egypt, Sudan, Morocco, Tunisia, Yemen), MSM account for a small proportion in reported HIV cases, which is most likely due to low likelihood of reporting a true mode of transmission in such cases due to stigma [2, 12–14]. Another explanation for the observed gender discrepancy is that men might be more frequently tested for HIV than women, in particular male migrant workers who are tested for HIV for the purpose of obtaining work permits in another country [14].

In 2011, only six countries (Jordan, Morocco, Oman, Palestine, Syria and Tunisia) were able to report data on CD4 counts in newly reported HIV cases at the time of HIV diagnosis, and in four of these data were considerably incomplete. In Jordan, Morocco, Oman, Palestine and Tunisia more than 50% of patients had CD4 counts <350 cells/mm^3^ at the time of diagnosis, which is to be expected considering rather low availability of HIV testing services [15].

Of note is that Somalia and Morocco that have some of the highest rates of reported HIV cases per 100,000 population do not have HIV mortality reporting. Reporting of HIV-related deaths through vital registration systems has proved to be challenging in many settings, including developed countries [16, 17]. In spite of that, efforts should be made in the Middle East and North Africa to strengthen AIDS deaths reporting, and systems that monitor effectiveness of HIV treatment and care programmes should be used as one of the tools for achieving this. WHO has recently launched a regional initiative To End the HIV Treatment Crisis, which aims to support countries in achieving a universal coverage of HIV treatment by 2020 [18]. In 2011 ART coverage in the region was only 12.5%, the lowest globally [19].

In conclusion, the coverage and validity of HIV case reporting vary among the countries, depending on the existence and accessibility of HIV testing services and reporting practices, and that creates a substantial challenge in interpreting HIV case reporting data. More efforts should be made in the countries to improve the validity of reported modes of HIV transmission and explore the reasons behind predominance of male HIV cases in the reported data. Better ascertainment of risk factor information is necessary, particularly in the countries of the Gulf Cooperation Council that report a high proportion of cases with unknown modes of HIV transmission. It is recommended that countries conduct periodic assessment of reporting to determine completeness, timeliness and error rates for the key variables, and if needed, provide training to health care staff to accurately record and report the data.

HIV case reporting, reporting of CD4 counts data where this diagnostics is available and reporting of HIV-related deaths should be strengthened as part of the on-going efforts to increase the access to HIV testing and treatment since they serve as tools to monitor and evaluate the progress made in delivering these interventions.
